# Associations of phthalates with NAFLD and liver fibrosis: A nationally representative cross-sectional study from NHANES 2017 to 2018

**DOI:** 10.3389/fnut.2022.1059675

**Published:** 2022-11-22

**Authors:** Xingying Chen, Feng Tian, Jianfeng Wu, Lan Liu, Ye Li, Genfeng Yu, Hualin Duan, Yuqi Jiang, Siyang Liu, Yajun He, Yaosheng Luo, Cheng Song, Huaizhi Li, Yongqian Liang, Heng Wan, Jie Shen

**Affiliations:** ^1^Department of Endocrinology and Metabolism, Shunde Hospital, Southern Medical University (The First People's Hospital of Shunde, Foshan), Foshan, China; ^2^Health Management Division, Shunde Hospital, Southern Medical University (The First People's Hospital of Shunde, Foshan), Foshan, China; ^3^Nantong Haimen People's Hospital, Haimen Hospital of Nantong University, Nantong, China; ^4^Shenzhen University General Hospital, Shenzhen University, Shenzhen, Guangdong, China

**Keywords:** phthalates, NAFLD, liver fibrosis, vibration-controlled transient elastography, NHANES (National Health and Nutrition Examination Survey)

## Abstract

**Objective:**

Although phthalates are common environmental pollutants, few studies have focused on the relationship of phthalates exposure with non-alcoholic fatty liver disease (NAFLD) or liver fibrosis, and especially, the alternative phthalates have been questioned in recent years about whether they are better choices. Thus, this study aimed to explore the associations of exposure to major phthalates or alternative phthalates with NAFLD and liver fibrosis.

**Methods:**

Data of 1450 adults from the National Health and Nutrition Examination Survey (NHANES) 2017-2018 were collected. The urinary metabolite concentrations of di-2-ethylhexyl phthalate (DEHP), diisononyl phthalate (DINP) and diisodecyl phthalate (DIDP) were detected. Controlled attenuation parameter (CAP) and median liver stiffness measurement (LSM) were acquired for quantitative diagnosis of NAFLD and liver fibrosis by vibration-controlled transient elastography. Multivariate logistic regression analysis and linear regression analysis were performed to examine the associations between phthalates and NAFLD and liver fibrosis.

**Results:**

After adjustment of the potential factors, the prevalence of NAFLD was significantly elevated among those in the fourth quartile of mono-(2-ethyl-5-carboxypentyl) phthalate (OR, 95%CI = 2.719, 1.296, 5.700, *P* = 0.016), mono (2-ethyl-5-hydroxyhexyl) phthalate (OR, 95%CI = 2.073, 1.111, 3.867, *P* = 0.037). No significant association was found between the alternative phthalates and NAFLD. The similar result was gained by linear regression analysis that MECPP was still significantly associated with Ln CAP (Q4 vs. Q1: β, 95%CI = 0.067, 0.017, 0.118, *P* = 0.027). After adjustment for the same covariates, no significant association between phthalates and liver fibrosis was found in logistics regression analysis.

**Conclusions:**

All in all, higher prevalence of NAFLD is correlated with DEHP but not DINP or DIDP in American adults. There is no significant relationship between phthalates and liver fibrosis defined as LSM ≥ 8 Kpa. Nevertheless, further research is needed to provide evidence of causality.

## Introduction

Non-alcoholic fatty liver disease (NAFLD) has become the most common chronic liver disease in the world. The prevalence of NAFLD is predicted to increase, and by 2030, NAFLD may affect 33.5% of the world's population aged ≥15 years (1). NAFLD encompasses a spectrum of conditions, from steatosis to non-alcoholic steatohepatitis, fibrosis and eventually cirrhosis, which is attributed mainly to insulin resistant, obesity, genetic variants and lifestyles (2, 3). However, in recent years, evidences reflect that exposure to some environmental contaminants may be considered as tangible risk factors for NAFLD (4, 5).

Phthalates, such as the major traditional plasticizers [e.g., di-2-ethylhexyl phthalate (DHEP)] and the alternative phthalates [e.g., diisononyl phthalate (DINP)], diisodecyl phthalate (DIDP), are used as plasticizers in numerous products. Due to the restriction of DEHP, the alternative phthalates such as DINP and DIDP have been widely used (6). They may become environmental pollutants since they can easily leach from products to environment (7). After absorbed by humans, phthalates are metabolized in the gastrointestinal tract and the liver, metabolites of phthalates such as MEHHP, MEOHP are finally excreted by urination (8). Thus, the urinary metabolite concentrations of phthalates are usually used for identifying phthalates exposure. As environmental endocrine-disrupting chemicals, phthalates are tightly associated with the prevalence of metabolic diseases, such as obesity and type 2 diabetes (9, 10). What's more, studies have showed that phthalates exposure to animal may interrupt lipid metabolism as well as induce inflammatory response in liver (11). However, the association of phthalates with NAFLD in humans is less clear. In addition, the association between phthalates and liver fibrosis is also unknown.

Two epidemiological studies have analyzed the relationship between exposure to traditional phthalates and NAFLD, in which NAFLD was defined by the US fatty liver index (US FLI) or the hepatic steatosis index (HSI) based on serum markers (12, 13). However, these serum biomarkers can be normal in patients with NAFLD and impacted by comorbid conditions, which may not be sufficiently sensitive in NAFLD definition and potentially underestimate the true population prevalence of NAFLD (14–16). Recently, some guidelines and expert consensus have recommended vibration-controlled transient elastography (VCTE) as a sensitive non-invasive tool in NAFLD evaluation especially in identifying advanced liver fibrosis (17, 18). VCTE, with high sensitivity and specificity of about 90%, can directly estimate the degree of hepatic steatosis and liver fibrosis with the controlled attenuation parameter (CAP) and liver stiffness measurement (LSM), respectively (19, 20).

Thus, in this study, we aimed to determine if the urinary metabolite levels of phthalates, including the alternative compounds, were associated with the prevalence of NAFLD and liver fibrosis detected by VCTE among American adults.

## Methods

### Study population

The National Health and Nutrition Examination Survey (NHANES) is a major program of the National Center for Health Statistics to assess health and nutritional status of civilians in America, which is approved by the Centers for Disease Control and Prevention Research Ethics Review Board. Both the physical examinations and blood collection were conducted in the mobile examination center (MEC), where laboratory tests were performed under standardized conditions. Demographic variables were collected and data were public. We analyzed data from NHANES 2017–2018, which can provide data on blood and urine phthalates exposure and VCTE.

**Figure 1:**
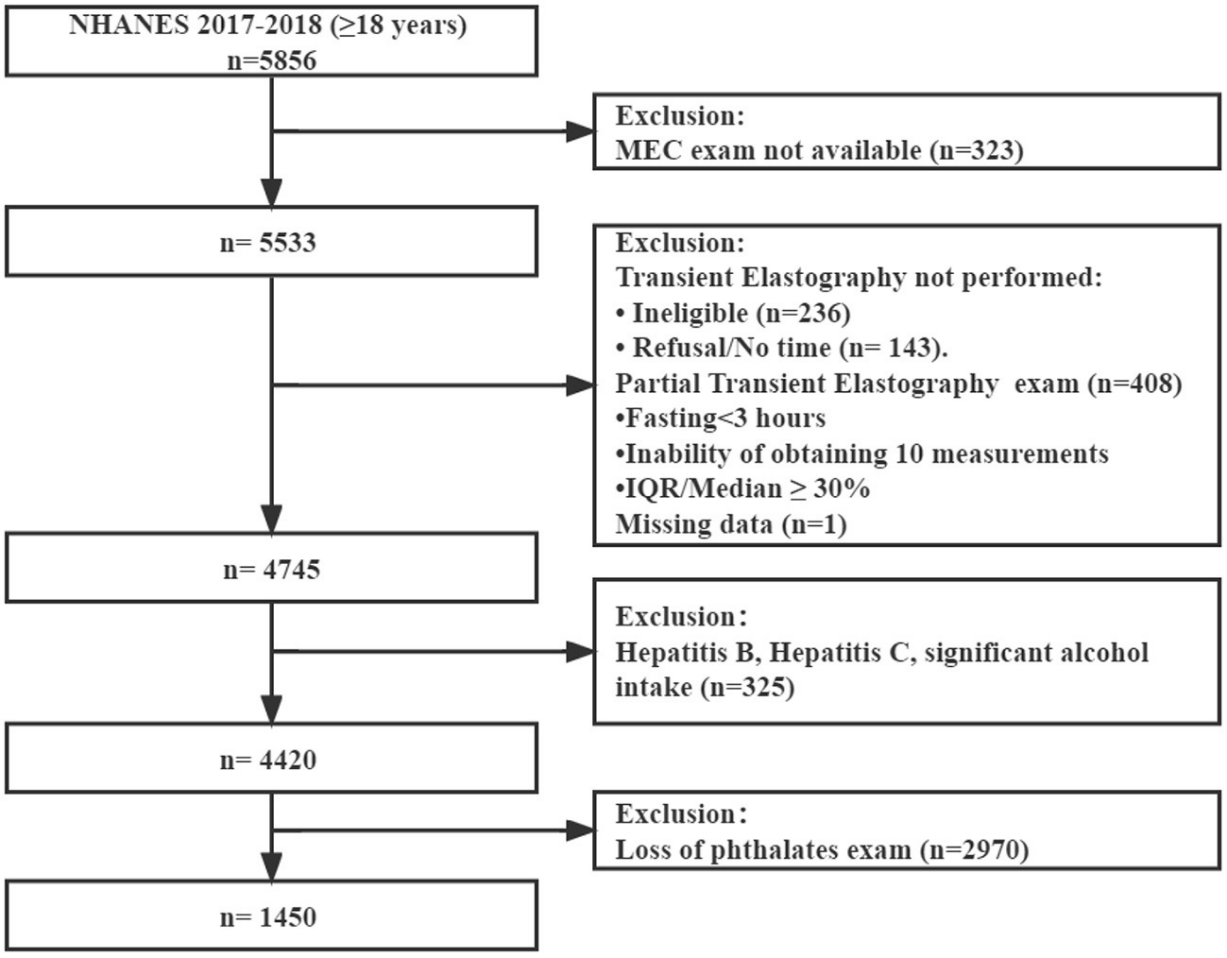
Data reduction diagram.

Initially, 5,856 participants aged 18 or older were included. First, 323 individuals considered to be not available for MEC exam were excluded. Second, 788 patients were excluded because of ineligible for the VCTE exam data and missing data. Third, 325 participants with hepatitis B or C or significant alcohol consumption (>30 g/day for men and >20 g/day for women) were excluded (21). Then, 2,970 participants who lacked data of urinary phthalates were excluded. Finally, 1,450 participants with complete data were enrolled (Figure 1).

### Measurements

#### Phthalates examination

We analyzed 6 metabolites of 3 phthalates (Supplementary Table S1): mono(2-ethyl-5-oxohexyl) phthalate (MEOHP), mono(2-ethyl-5-hydroxyhexyl) phthalate (MEHHP), mono-(2-ethyl-5-carboxypentyl) phthalate (MECPP), mono-(carboxyoctyl) phthalate (MCiOP), mono-(carboxynonyl) phthalate (MCiNP) and mono-oxoisononyl phthalate (MOiNP). High-performance liquid chromatography-electrospray ionization-tandem mass spectrometry (HPLC-ESI-MS/MS), and online solid phase extraction coupled with isotope dilution-HPLC-MS/MS were used to quantitatively detect the urine levels of the 6 metabolites. Samples were assigned with the limit of detection (LOD) divided by the square root of two as recommended by NCHS when their concentrations were below the LOD. Detailed contents of analysis methods can be queried on the website (https://wwwn.cdc.gov/nchs/nhanes/analyticguidelines.aspx).

#### Definition of NAFLD and liver fibrosis

The CAP and LSM, which reflect liver steatosis and fibrosis, respectively, were assessed using VCTE. VCTE was performed by the trained technicians using a FibroScan model 502 V2 Touch (Echosens, Paris, France). The medium probe was applied firstly. If recommend by the manufacturer's instructions, an XL-probe was used instead of M-probe. Examinations were considered reliable if (i) participants fasted at least 3 h before the exam, (ii) 10 or more complete LSMs were performed, and (iii) the interquartile range/median of LSM was < 30%. Detailed procedure was recorded in the Liver Ultrasound Transient Elastography Procedures Manual. We defined NAFLD as CAP ≥274 dB/m and LSM ≥8 kPa as a threshold for liver fibrosis according to the literature (22).

In addition, we also calculated HSI and US FLI. In this study, NAFLD was defined as HSI > 36 and the cut-off of US FLI to diagnose NAFLD is 30 or higher (13). The HSI and US FLI were calculated as follows:

HSI = 8 ^*^ ALT (IU/L)/AST (IU/L) + body mass index (BMI, kg/m^2^) +2 (if female) +2 (if type 2 diabetes).

US FLI = (e^−0.8073 * non-Hispanic black + 0.3458 * Mexican American + 0.0093 * age + 0.6151 * loge (GGT) + 0.0249 * waist circumference + 1.1792 * loge (insulin) + 0.82 * loge (glucose) − 14.7812^) / (1 + e^−0.8073 * non-Hispanic black + 0.3458 * Mexican American + 0.0093 * age + 0.6151 * loge (GGT) + 0.0249* waist circumference + 1.1792 * loge (insulin) + 0.8242* loge (glucose) −14.7812^) * 100

#### Covariates definition

Other covariates included sociodemographic variables, such as age, sex (male or female), smoking status (< 100, 100 or more), education (high school degree and below, higher than high school) and race/ethnicity (Mexican American, non-Hispanic white, non-Hispanic black, or other races). Physical activity (PA) was further dichotomized into achieved PA or insufficient in line with the 2018 Physical Activity Guidelines Advisory Committee Scientific Report. Diabetes was determined using a previous diagnosis by healthcare professionals according to fasting plasma glucose (FPG) level ≥7.0 mmol/L or HbA1c ≥6.5%, or self-reported diabetes diagnosis. Blood pressure (BP) was measured three times by trained nurses and the average value was used for subsequent analysis. Covariates were also collected such as body mass index (BMI) and total cholesterol levels.

### Statistical analysis

Data were analyzed using IBM SPSS Statistics 26 (IBM Corporation, Armonk, NY, USA) and software R 4.1.1. Benjamini-Hochberg false discovery rate was performed to correct *P*-values for multiple comparisons and two-tailed *P* < 0.05 was considered significant. The proper weight was used because of the complex design of NHANES. Continuous variables were described as means ± standard error (SE) and analyzed using *t*-test. Categorical variables were expressed as numbers (percent) and compared by the chi-square test. The levels of urinary phthalate metabolites were corrected by the creatinine level in μg/g to account for differences in kidney function. Due to the skewed distribution, the levels of phthalate metabolites were further transformed by a natural logarithm. Quartiles of urinary phthalate metabolite concentrations were treated as exposure variables for subsequent analyses.

Multivariate logistic regression analysis was performed to analyze the associations of the prevalence of NAFLD and liver fibrosis with phthalates after accounting for potential confounders. The model was adjusted for age, gender, race, BMI, total cholesterol level, systolic blood pressure, educational level, smoking status, diabetes status and physical activity. In addition, CAP and LSM were ln-transformed to calculate the significance determined by a linear regression model after adjusting for the same underlying confounders as well. Finally, we repeated the multivariate logistic regression analysis to estimate the association between phthalates and NAFLD defined by US FLI or HSI.

## Results

### Population characteristics

Table 1. Totally 1,450 participants with an average age of 46 years were included. Among them, the numbers of men and women were almost the same (50.1 vs. 49.9%). Most of them were white and had received higher than high school education. The majority of the participants had achieved guidelines for PA and no diabetes. The proportion of smokers was less than that of non-smokers.

**Table 1:** Population characteristics of American adults with or without NAFLD from NHANES 2017–2018.

**Characteristic**	**Total (*N* = 1,450)**	**NAFLD**		***P*-value**
		**No**	**Yes**	
		**(*N* = 837)**	**(*N* = 613)**	
Age, y	46.2 ± 0.81	43.2 ± 1.03	50.7 ± 0.92	**< 0.001**
Gender, %				**0.015**
Male	50.1	46.4	55.5	
Female	49.9	53.6	44.5	
Race, %				**< 0.001**
Mexican American	9.4	6.8	13.4	
Non-Hispanic White	60.7	61.3	59.7	
Non-Hispanic Black	11.3	12.9	9.1	
Other race	18.6	19.1	17.9	
Physical activity, %				**< 0.001**
Insufficient	31.7	26.9	38.9	
Achieved PA	68.3	73.1	61.1	
Education, %				0.650
≤ High school	39.0	38.1	40.1	
>High school	61.0	61.8	59.9	
Current smoking, %	0.572
No	85.9	85.4	86.6	
Yes	14.1	14.6	13.4	
Diabetes, %				**< 0.001**
No	77.2	88.6	62.6	
Yes	22.8	11.4	37.4	
SBP, mmHg	120.6 ± 0.49	118.6 ± 0.63	123.5 ± 0.96	**0.001**
BMI, kg/m^2^	29.2 ± 0.31	26.3 ± 0.36	33.5 ± 0.38	**< 0.001**
FPG, mmol/L	6.1 ± 0.08	5.7 ± 0.05	6.6 ± 0.18	**< 0.001**
HbA1c, %	5.6 ± 0.02	5.5 ± 0.02	5.9 ± 0.05	**< 0.001**
TC, mmol/L	4.9 ± 0.05	4.9 ± 0.06	5.0 ± 0.07	0.409
ALT, U/L	22.6 ± 0.72	20.1 ± 0.84	26.3 ± 1.14	**< 0.001**
AST, U/L	21.5 ± 0.49	21.2 ± 0.71	22.0 ± 0.53	0.339
GGT, U/L	27.4 ± 0.99	23.0 ± 1.36	33.8 ± 1.42	**< 0.001**
The ln-transferred of urinary phthalate metabolites, μg/g Cr				
MECPP	1.89 ± 0.03	1.84 ± 0.03	1.97 ± 0.05	**0.012**
MEOHP	0.99 ± 0.03	0.95 ± 0.03	1.07 ± 0.05	**0.028**
MEHHP	1.44 ± 0.03	1.40 ± 0.03	1.50 ± 0.05	0.084
MCiNP	0.16 ± 0.02	0.15 ± 0.03	0.17 ± 0.04	0.738
MCiOP	1.51 ± 0.04	1.51 ± 0.04	1.52 ± 0.05	0.749
MOiNP	0.19 ± 0.03	0.20 ± 0.03	0.18 ± 0.05	0.790

Data are expressed as numbers (percent) for categorical variables and as means ± standard error (SE) for continuous variables.

BMI, body mass index; FPG, fasting plasma glucose; HbA1C, glycohemoglobin; ALT, Alanine aminotransferase; AST, Aspartate aminotransferase; GGT, γ-glutamyl transpeptidase; SBP, systolic blood pressure; NHANES, National Health and Nutrition Examination Survey. *P* values are bolded when *P* < 0.05. (The same in Table 2).

Clinical features of the patients with or without NAFLD were also shown in Table 1. In total, 613 patients were diagnosed NAFLD (defined by the cut-off CAP of ≥ 274 dB/m, weighted prevalence 40.2%). Compared to the subjects without NAFLD, those with NAFLD were more often male and likely to be older (50.7 vs. 43.2 years old), having larger BMI (33.5 vs. 26.3), and diabetic (37.4 vs. 11.4%). The subjects with NAFLD also had higher levels of systolic blood pressure, FPG, alanine aminotransferase (ALT), glutamine transferase (GGT), HbA1c and urinary concentrations of MECPP and MEOHP. In addition, significant baseline differences in the distributions of race/ethnicity and physical activity existed between these two groups.

Table 2 showed the clinical characteristics of the patients, stratified by liver fibrosis (defined by the cut-off LSM of ≥8 Kpa, weighted prevalence 6.7%). Significant fibrosis was found in 116 patients. Similarly, participants with liver fibrosis were older, and commonly had higher BMI. They showed a higher prevalence of diabetes and did not achieve the physical activity guidelines. As for serum indexes, patients with liver fibrosis had a higher fasting blood glucose, HbA1c and worst liver conditions with higher ALT, AST, and GGT levels.

**Table 2:** Population characteristics of American adults with or without liver fibrosis from NHANES 2017–2018.

**Characteristic**	**Total**	**Liver fibrosis**		***P*-value**
	**(*N* = 1,450)**	**No**	**Yes**	
		**(*N* = 1,334)**	**(*N* = 116)**	
Age, y	46.2 ± 0.81	45.7 ± 0.85	52.9 ± 2.39	**0.013**
Gender, %				0.415
Male	50.1	49.7	55.8	
Female	49.9	50.3	44.2	
Race, %				0.396
Mexican American	9.4	9.3	11.2	
Non-Hispanic White	60.7	61.1	54	
Non-Hispanic Black	11.3	11.1	14.6	
Other Race	18.6	18.5	20.2	
Physical activity, %				**< 0.001**
Insufficient	31.7	29.9	56.6	
Achieved PA	68.3	70.1	43.4	
Education, %				**0.001**
≤ High school	39.0	38	52.9	
>High school	61.0	62	47.1	
Current smoking, %	
No	85.9	85.8	86.9	0.817
Yes	14.1	14.2	13.1	
Diabetes, %	
No	77.2	81.3	34.9	**< 0.001**
Yes	22.8	18.7	65.1	
SBP, mmHg	120.6 ± 0.49	120.4 ± 0.57	122.5 ± 1.38	0.243
BMI, kg/m^2^	29.2 ± 0.31	28.5 ± 0.36	38.2 ± 0.60	**< 0.001**
FPG, mmol/L	6.1 ± 0.08	5.9 ± 0.07	7.2 ± 0.36	**0.002**
HbA1c, %	5.6 ± 0.02	5.5 ± 0.02	6.4 ± 0.13	**< 0.001**
TC, mmol/L	4.9 ± 0.05	4.9 ± 0.04	4.7 ± 0.16	0.189
ALT, U/L	22.6 ± 0.72	21.9 ± 0.72	32.7 ± 2.45	**< 0.001**
AST, U/L	21.5 ± 0.49	21.0 ± 0.46	28.1 ± 2.30	**0.005**
GGT, U/L	27.4 ± 0.99	24.8 ± 0.89	62.9 ± 8.09	**< 0.001**
The ln-transferred of urinary phthalate metabolites, μg/g Cr				
MECPP	1.89 ± 0.03	1.89 ± 0.03	2.00 ± 0.07	0.057
MEOHP	0.99 ± 0.03	0.99 ± 0.03	1.04 ± 0.08	0.552
MEHHP	1.44 ± 0.03	1.44 ± 0.03	1.49 ± 0.09	0.537
MCiNP	0.16 ± 0.02	0.15 ± 0.02	0.24 ± 0.06	0.204
MCiOP	1.51 ± 0.04	1.51 ± 0.04	1.50 ± 0.07	0.881
MOiNP	0.19 ± 0.03	0.19 ± 0.03	0.25 ± 0.08	0.543

*P* values are bolded when *P* < 0.05.

### Associations of urinary phthalate metabolites with NAFLD and liver fibrosis

The associations between the higher levels of phthalates and the prevalence of NAFLD (defined as CAP ≥274 dB/m) were reported in Table 3. The logistics regression analysis was adjusted for age, gender, race, BMI, total cholesterol level, systolic blood pressure, educational level, smoking status, diabetes status and physical activity. The logistics regression analysis showed that individuals in the higher quartile of MECPP had higher prevalence of NAFLD (Q4 vs. Q1: OR = 2.719, 95%CI: 1.296, 5.700, *P* = 0.016; Q2 vs. Q1: OR = 2.277, 95%CI: 1.277, 4.060, *P* = 0.016) compared to the first quartile. The prevalence of NAFLD was also significantly associated with elevated MEHHP levels (Q4 vs. Q1: OR = 2.073, 95%CI: 1.111, 3.867, *P* = 0.037; Q3 vs. Q1: OR = 1.816, 95%CI: 1.106, 2.981, *P* = 0.037; Q2 vs. Q1: OR = 2.110, 95%CI: 1.009, 4.409, *P* = 0.047). When the association was analyzed by general linear regression, the similar results were found that MECPP was still significantly associated with Ln CAP (Q4 vs. Q1: β = 0.06, 95%CI: 0.017, 0.118, *P* = 0.027; Q2 vs. Q1: β = 0.044, 95%CI: 0.008, 0.079, *P* = 0.027). When NAFLD was defined as HSI >36 or US FLI ≥30, no significant association was found between phthalates and NAFLD (Supplementary Table S2). Participants did not show a significant increase in the prevalence of liver fibrosis when considering the increasing urinary phthalate metabolites concentrations. The third quartile of MCiNP and MCiOP were correlated with LSM in the results of the linear regression tests (*P* = 0.009, 0.006, respectively) (Table 4).

**Table 3:** The associations between phthalates and NAFLD in US adults.

**Phthalate**	**NAFLD**		**Ln CAP**	
**metabolites**	**(CAP** ≥**274 dB/m)**			
	**OR (95%CI)**	***P*_FDR_**	**β (95%CI)**	***P*_FDR_**
MECPP				
Q1	Ref.		Ref.	
Q2	**2.277 (1.277, 4.06)**	**0.016**	**0.044 (0.008, 0.079)**	**0.027**
Q3	1.435 (0.663, 3.107)	0.335	0.007 (−0.043, 0.057)	0.777
Q4	**2.719 (1.296, 5.700)**	**0.016**	**0.067 (0.017, 0.118)**	**0.027**
MEOHP				
Q1	Ref.		Ref.	
Q2	1.662 (0.999, 2.764)	0.068	0.021 (−0.025, 0.067)	0.510
Q3	1.452 (0.967, 2.179)	0.068	0.006 (−0.049, 0.060)	0.826
Q4	2.016 (1.102, 3.687)	0.068	0.036 (−0.005, 0.076)	0.243
MEHHP				
Q1	Ref.		Ref.	
Q2	**2.110 (1.009, 4.409)**	**0.047**	0.020 (−0.049, 0.089)	0.552
Q3	**1.816 (1.106, 2.981)**	**0.037**	0.051 (0.008, 0.094)	0.072
Q4	**2.073 (1.111, 3.867)**	**0.037**	0.046 (−0.002, 0.094)	0.085
MCiNP				
Q1	Ref.		Ref.	
Q2	1.662 (1.013, 2.728)	0.134	0.034 (−0.025, 0.093)	0.415
Q3	1.558 (0.788, 3.081)	0.185	0.024 (−0.040, 0.089)	0.433
Q4	1.618 (0.917, 2.854)	0.136	0.031 (−0.027, 0.089)	0.415
MCiOP				
Q1	Ref.		Ref.	
Q2	0.976 (0.567, 1.678)	0.925	−0.027 (−0.078, 0.023)	0.403
Q3	2.409 (1.028, 5.643)	0.108	0.070 (0.011, 0.130)	0.072
Q4	1.645 (0.950, 2.847)	0.108	0.022 (−0.033, 0.077)	0.408
MOiNP				
Q1	Ref.		Ref.	
Q2	1.266 (0.485, 3.299)	0.607	0.003 (−0.053, 0.058)	0.923
Q3	1.901 (0.871, 4.146)	0.298	0.045 (−0.002, 0.092)	0.177
Q4	1.556 (0.643, 3.766)	0.454	0.022 (−0.025, 0.069)	0.499

Data are expressed as odds ratio (95% Confidence interval [CI]) or β (95% Confidence interval [CI]). Odds ratios (95% CIs) were adjusted for age, gender, race.

BMI, total cholesterol level, systolic blood pressure, educational level, smoking status, diabetes status and physical activity.

NHANES, National Health and Nutrition Examination Survey. All *P*-values were corrected by FDR (the same in Table 4).

**Table 4:** The associations between phthalates and liver fibrosis in US adults.

**Phthalate**	**Fibrosis (LSM** ≥**8 Kpa)**		**Ln LSM**	
**metabolites**	
	**OR (95%CI)**	***P*_FDR_**	**β (95%CI)**	***P*_FDR_**
MECPP				
Q1	Ref.		Ref.	
Q2	1.316 (0.304, 5.692)	0.695	−0.006 (−0.115, 0.103)	0.903
Q3	3.284 (0.995, 10.841)	0.153	0.072 (−0.054, 0.197)	0.531
Q4	0.697 (0.220, 2.214)	0.695	−0.033 (−0.106, 0.04)	0.531
MEOHP				
Q1	Ref.		Ref.	
Q2	0.971 (0.442, 2.131)	0.938	−0.025 (−0.105, 0.054)	0.760
Q3	1.130 (0.390, 3.273)	0.938	0.001 (−0.112, 0.114)	0.981
Q4	0.536 (0.129, 2.218)	0.938	−0.068 (−0.145, 0.008)	0.228
MEHHP				
Q1	Ref.		Ref.	
Q2	1.679 (0.592, 4.758)	0.458	0.006 (−0.071, 0.084)	0.864
Q3	1.660 (0.670, 4.115)	0.458	−0.032 (−0.128, 0.064)	0.864
Q4	0.595 (0.110, 3.207)	0.521	−0.028 (−0.151, 0.095)	0.864
MCiNP				
Q1	Ref.		Ref.	
Q2	0.784 (0.262, 2.350)	0.645	0.048 (−0.045, 0.141)	0.429
Q3	1.871 (0.576, 6.074)	0.411	**0.109 (0.042, 0.176)**	**0.009**
Q4	1.654 (0.700, 3.905)	0.411	0.042 (−0.081, 0.165)	0.476
MCiOP				
Q1	Ref.		Ref.	
Q2	0.602 (0.149, 2.418)	0.673	0.046 (−0.065, 0.157)	0.392
Q3	1.987 (0.764, 5.167)	0.438	**0.11 (0.046, 0.174)**	**0.006**
Q4	0.949 (0.254, 3.541)	0.935	0.036 (−0.05, 0.122)	0.392
MOiNP				
Q1	Ref.		Ref.	
Q2	2.861 (0.587, 13.93)	0.265	0.099 (0.005, 0.193)	0.123
Q3	3.611 (0.793, 16.42)	0.265	0.067 (−0.036, 0.17)	0.277
Q4	1.589 (0.588, 4.291)	0.336	0.047 (−0.058, 0.151)	0.357

## Discussion

In the current study, the results showed that higher urinary concentrations of phthalate metabolites that metabolized from DEHP, were positively related to NAFLD. No significant association was observed between phthalates and liver fibrosis defined as LSM ≥8 Kpa. As far as we know, this is the first study to investigate the relationships of phthalate exposure with NAFLD and liver fibrosis assessed by VCTE.

Fewer epidemiologic studies explored the relationship of phthalates with NAFLD diagnosed by serum biomarkers in different races. An earlier study including 5,800 Korea adults indicated that the higher quartiles of MEHHP showed significant higher ORs of NAFLD defined by HSI (12). Recently, another cross-sectional study with 4,206 American subjects demonstrated that DEHP metabolites exposure was independently associated with NAFLD defined by HSI and results maintained when defined as US FLI (13). In accordance with the two above studies, our research showed similar positive associations of MECPP and MEHHP with NAFLD measured by VCTE. Moreover, MECPP exposure was still significant associated with Ln CAP, which suggests the metabolite may be associated with the severity of NAFLD. Inconsistent with above studies, the present study showed that no significant association was found between phthalates and NAFLD when NAFLD was defined as HSI >36 or US FLI ≥30 in our study. This may be accounted for AST levels are similar between patients with NAFLD or without NAFLD and these indexes are not sensitive enough, whereas liver VCTE are potentially more sensitive (23). What's more, the different covariates in these studies may be partially attributed to the inconsistent results. Laboratory studies indicated that DEHP and its metabolite MEHP may interfere liver lipid metabolism to induce NAFLD (24, 25). Analysis of underlying mechanisms showed that DEHP may cause lipid metabolism disorder through hepatic PPAR, the main protein of metabolic homeostasis regulation, and upregulate DGAT1, the key enzyme responsible for synthesis and storage of TGs in the liver (26–28).

Another potential mechanism needs our attention is that thyroid function may mediate the relationship of DEHP and NAFLD. Thyroid hormones' role of mediator in the associations between phthalate exposure and lipid metabolism has been reported (29). A recent study demonstrated that positive association between the urine levels of phthalate metabolites and NAFLD was observed in adults with subclinical hypothyroidism, but not in those with euthyroidism (30). In fact, evidence of a higher prevalence of NAFLD in patients with hypothyroidism has been detailed recorded in previous literature (23, 31). Thyroid hormones mainly activate TH-Receptor β, a potential target in NAFLD therapy, and thus may improve liver steatosis (32, 33). DEHP may lead to thyroid function disorder since DEHP possessed thyroid receptor (TR) antagonist activity (34). Therefore, DEHP may interfere thyroid function and further induce NAFLD. However, the associations between DEHP and thyroid hormones were seen not in all studies (35). Thus, the complex relationships between DEHP, thyroid hormones and NAFLD should be further investigated.

Compared with the major phthalate DEHP, the associations of the alternative compounds DINP and DIDP with NAFLD have not been studied yet. Our research showed no significant relationship between NAFLD and DIDP or DINP. Although some studies demonstrated that DINP and DIDP could disturb lipid metabolism in fish, which raised the concern about the environmental exposure to these alternatives as a possible contributor (36, 37). Furthermore, Yang et al. reported that DINP induced a greater alteration of lipidomic markers for hepatic steatosis than DEHP in post-weaning mice, which may contribute to the etiology of NAFLD (38). Further study is needed.

Nonetheless, the results about a positive association of DEHP, but not the alternative compounds, with NAFLD may be accounted for higher energy intake in patients with NAFLD, data of which were not collected in our dataset. Patients with NAFLD have larger BMI in our study, who may have higher energy intake. Phthalates exposure, especially DEHP but not DiNP, was contributed from food to some extent (39). Thus, patients with NAFLD may have higher energy intake and a higher intake of DEHP rather than DINP. Therefore, further studies are required to investigate the possibility.

Meanwhile, our study found significant relationship between phthalates and the prevalence of liver fibrosis was not clearly observed. In fact, some experimental studies had tried to explore the relationship between phthalates and liver fibrosis, and found the liver toxicity of phthalates in animals through oxidative stress pathways, which may drive liver inflammation and fibrosis (40–42). Lee et al. also illustrated that long-term exposure to DEHP may perturb the cholesterol metabolism in HSCs and thus accelerate liver damage and fibrosis (43). More studies are warranted to reveal the association of phthalates and liver fibrosis. Although our data showed that risk of liver fibrosis may not increase in individuals with higher phthalate exposure, caution is required due to the small number of participants with fibrosis in our dataset.

There are some limitations in our study. Firstly, since this is a cross-sectional study, we cannot confirm the causal relationship between phthalates and NAFLD. Therefore, cohort studies or case-control studies should be done to overcome this methodological limitation. Secondly, regardless of the many benefits, VCTE is not a gold standard technique. Although liver biopsy is the gold standard for diagnosing fatty liver, it is hard to include adequate patients in population studies. Other non-invasive measures to define NAFLD such as HSI or plasma biochemical indicators is easy to gain but may be insufficient sensitive in NAFLD diagnosis. VCTE has its flaws, which includes operator dependent accuracy and limited by body habitus/ascites. However, VCTE were conducted by trained NHANES health technicians to ensure results as accurate as possible. Finally, urinary phthalates were measured only in a one third subsample of participants and thus a number of participants were excluded from this analysis. In a recent study using data from the NHANES 2017–2018, the prevalence of NAFLD was 37%, which is comparable to the findings in this study (44). In addition, the prevalence of liver fibrosis reported here is lower than the prevalence estimates of a recent research, which may be accounted for the different target populations (45).

## Conclusions

An independent association of the prevalence of NAFLD with DEHP metabolites exposure, but not the alternative phthalates, was found in American adults. Further case-control studies or longitudinal cohort studies are needed to reveal their causal relationship.

## Data availability statement

Publicly available datasets were analyzed in this study. This data can be found here: https://www.cdc.gov/nchs/nhanes/index.htm.

## Ethics statement

The studies involving human participants were reviewed and approved by the study protocol was approved by the NCHS Research Ethics Review Board. Written informed consent for participation was not required for this study in accordance with the national legislation and the institutional requirements.

## Author contributions

XC, FT, and HW designed the study. LL, YeL, GY, HD, YJ, YH, YaL, CS, HL, and FT collected the data. YeL, JW, and YJ validated the data. XC, LL, SL, and FT analyzed the data. XC, FT, and JW wrote the manuscript. HW, YoL, and JS revised the manuscript and supervised the study. All authors read and approved the final manuscript.

## Funding

This study was supported by the National Natural Science Foundation of China (82170800); Guangdong Basic and Applied Basic Research Foundation (2021A1515110682); Start of clinical research of Southern Medical University (IC2016PY046); Research Initiation Project of Shunde Hospital of Southern Medical University (SRSP2021001). The funders played no role in the design or conduct of the study, collection, management, analysis, interpretation of data or in the preparation, review, and approval of the article.

## Conflict of interest

The authors declare that the research was conducted in the absence of any commercial or financial relationships that could be construed as a potential conflict of interest.

## Publisher's note

All claims expressed in this article are solely those of the authors and do not necessarily represent those of their affiliated organizations, or those of the publisher, the editors and the reviewers. Any product that may be evaluated in this article, or claim that may be made by its manufacturer, is not guaranteed or endorsed by the publisher.
