# Supplementary material for: Associations of phthalates with NAFLD and liver fibrosis: A nationally representative cross-sectional study from NHANES 2017 to 2018
Source: Front Nutr. 2022 Nov 22;9:1059675. doi: 10.3389/fnut.2022.1059675 (PMC9723339; doi:10.3389/fnut.2022.1059675)
Supplement: Supplementary file 1 [file Data_Sheet_1.docx]

**Table S1.** Parent Compounds of Phthalates and their Major Metabolites

| Parent Compounds | Abb. | Major Metabolites | Abb. |
| --- | --- | --- | --- |
| Di-2-ethylhexyl phthalate | DEHP | Mono-2-ethyl-5-carboxypentyl phthalate | MECPP |
|  |  | Mono-(2-ethyl-5-hydroxyhexyl) phthalate | MEHHP |
|  |  | Mono-(2-ethyl-5-oxohexyl) phthalate | MEOHP |
| Diisodecyl phthalate | DiDP | Mono(carboxyisononyl) phthalate | MCiNP |
| Diisononyl phthalate | DiNP | Mono-oxoisononyl phthalate | MOiNP |
|  |  | Mono(carboxyisoctyl) phthalate | MCiOP |

Abb.: abbreviation.

**Table S2.** The associations between phthalates and NAFLD (defined by HSI or US FLI) in US adults

| phthalate metabolites | NAFLD (HSI>36) | |  | NAFLD (US FLI≥30) | |
| --- | --- | --- | --- | --- | --- |
|  | OR (95%CI) | *P* _FDR_ |  | OR (95%CI) | *P* _FDR_ |
| MECPP |  |  |  |  |  |
| Q1 | Ref. |  |  | Ref. |  |
| Q2 | 2.996(0.974,9.212) | 0.164 |  | 1.327(0.234,7.528) | 0.733 |
| Q3 | 0.411(0.086,1.952) | 0.364 |  | 0.729(0.181,2.936) | 0.733 |
| Q4 | 0.751(0.125,4.496) | 0.738 |  | 1.421(0.331,6.097) | 0.733 |
| MEOHP |  |  |  |  |  |
| Q1 | Ref. |  |  | v |  |
| Q2 | 2.218(0.561,8.759) | 0.653 |  | 0.895(0.202,3.970) | 0.894 |
| Q3 | 1.879(0.350,10.07) | 0.653 |  | 0.804(0.194,3.325) | 0.894 |
| Q4 | 0.881(0.126,6.158) | 0.892 |  | 0.919(0.247,3.420) | 0.894 |
| MEHHP |  |  |  |  |  |
| Q1 | Ref. |  |  | Ref. |  |
| Q2 | 1.430(0.278,7.354) | 0.907 |  | 0.916(0.242,3.456) | 0.962 |
| Q3 | 1.088(0.236,5.005) | 0.907 |  | 1.030(0.274,3.868) | 0.962 |
| Q4 | 0.630(0.088,4.500) | 0.907 |  | 0.880(0.205,3.765) | 0.962 |
| MCiNP |  |  |  |  |  |
| Q1 | Ref. |  |  | Ref. |  |
| Q2 | 2.059(0.292,14.50) | 0.948 |  | 0.317(0.053,1.891) | 0.572 |
| Q3 | 1.048(0.224,4.907) | 0.948 |  | 0.685(0.150,3.110) | 0.825 |
| Q4 | 1.091(0.349,3.409) | 0.948 |  | 0.868(0.227,3.319) | 0.825 |
| MCiOP |  |  |  |  |  |
| Q1 | Ref. |  |  | Ref. |  |
| Q2 | 1.083(0.182,6.430) | 0.925 |  | 0.783(0.321,1.913) | 0.673 |
| Q3 | 0.899(0.131,6.140) | 0.925 |  | 1.255(0.407,3.864) | 0.673 |
| Q4 | 0.792(0.192,3.260) | 0.925 |  | 0.557(0.207,1.499) | 0.673 |
| MOiNP |  |  |  |  |  |
| Q1 | Ref. |  |  | Ref. |  |
| Q2 | 0.613(0.129,2.917) | 0.515 |  | 0.441(0.127,1.532) | 0.273 |
| Q3 | 0.617(0.188,2.018) | 0.515 |  | 1.071(0.369,3.105) | 0.892 |
| Q4 | 0.547(0.149,2.008) | 0.515 |  | 0.333(0.115,0.964) | 0.131 |

Data are expressed as odds ratio (95% Confidence interval [CI]). Odds ratios (95% CIs) were adjusted for age, gender, race, BMI, total cholesterol level, systolic blood pressure, educational level, smoking status, diabetes status and physical activity. NHANES: National Health and Nutrition Examination Survey. All *P* values were corrected by FDR
